# Intestinal anti-inflammatory and visceral analgesic effects of a *Serpylli herba* extract in an experimental model of irritable bowel syndrome in rats

**DOI:** 10.3389/fphar.2022.967644

**Published:** 2022-09-02

**Authors:** Antonio Jesús Ruiz-Malagón, María José Rodríguez-Sanchez, María Jesús Rodríguez-Sojo, Teresa Vezza, Ivo Pischel, Francesca Algieri, María Elena Rodríguez-Cabezas, Alba Rodríguez-Nogales, Julio Gálvez

**Affiliations:** ^1^ Center for Biomedical Research (CIBM), Department of Pharmacology, University of Granada, Granada, Spain; ^2^ Instituto de Investigación Biosanitaria de Granada (ibs.GRANADA), Granada, Spain; ^3^ Servicio de Digestivo, Hospital Universitario Virgen de las Nieves, Granada, Spain; ^4^ Centre for Pharmacognosy and Phytotherapy, UCL School of Pharmacy, University of London, London, United Kingdom; ^5^ Centro de Investigación Biomédica en Red de Enfermedades Hepáticas y Digestivas (CIBEREHD), Madrid, Spain

**Keywords:** *Serpylli herba*, *T. serpyllum* L., intestine anti inflammatory activity, visceral analgesia, IBS rat model

## Abstract

**Ethnopharmacological relevance:** *Serpylli herba* extract (SHE), composed of the aerial parts of wild thyme (*Thymus serpyllum* L.) (*Lamiaceae* family), is traditionally used in Europe and North Africa to treat diarrhea, gastric ulcers, intestinal parasites and upper respiratory tract infections. Recently, SHE has generated a great interest for irritable bowel syndrome (IBS) management, probably due to its intestinal anti-inflammatory properties shown in experimental colitis and the fact that its active components could preserve the intestinal barrier integrity, which is altered in patients with IBS.

**Aim of study:** We aimed to test the effects of a SHE in a rat experimental model resembling human IBS.

**Materials and methods:** IBS was provoked by deoxycholic acid (DCA). Rats were then treated with SHE (100 mg/kg) or gabapentin (70 mg/kg) and different inflammatory and gut barrier integrity markers were evaluated. Moreover, several gut hypersensitivity and hyperalgesia determinations were performed.

**Results:** SHE improved referred pain and visceral hypersensitivity. Additionally, SHE enhanced immune status by downregulating of the expression of the pro-inflammatory mediators Il-1β, Il-6, Ifn-γ, Tlr-4, and the inducible enzyme *Cox-2*, thus inducing visceral analgesia, and promoting the restore of the gut barrier function by upregulating the mucins *Muc-2* and *Muc-3*. These anti-inflammatory effects could be related to its action on mast cells since it significantly inhibited the *β*-Hexosaminidase production in RBL-2H3 cells. Lastly, SHE also seems to modulate the serotonin pathway by restoring the altered expression of the 5-HT receptors *Htr-3* and *Htr-4*.

**Conclusion:** SHE could be considered a potential new treatment for IBS, since it ameliorates hypersensitivity, visceral hyperalgesia, and inflammation. These beneficial effects may be due to the inhibition of mast cells degranulation and serotonin pathway.

## Introduction

IBS is a very prevalent functional digestive disease, being its worldwide prevalence of 11% (Mearin et al., 2016). It is distinguished by recurrent abdominal pain, bloating, cramping, gas and altered bowel habits as per the diagnostic Rome IV criteria (Mearin et al., 2016; Weaver et al., 2017). Moreover, 50%–90% of the patients present psychiatric comorbidities, including anxiety, social phobia, somatization disorder, major depressive disorder, or posttraumatic stress (Banerjee et al., 2017). Its etiology and pathophysiology are not properly understood, but gastrointestinal dysmotility, compromised gut barrier function, altered intestinal immune activation and visceral hypersensitivity are associated with its development (Lee and Park, 2014). Indeed, it has been described that a persistently augmented presence of intraepithelial lymphocytes and enteroendocrine and mast cells in the intestinal mucosa unbalance the network that links the enteric immune defense and the nervous system and leads to the onset of IBS symptoms (Yoo and Mazmanian, 2017; Uranga et al., 2020). Particularly, the sensitization of the immune cells triggers the release of cytokines and mediators that can affect different motor and neural functions, and produce pain, irritability, and hypersensitivity (Lee and Park, 2014; Uranga et al., 2020).

To date, the management of this disorder is limited because the drugs that are used are not well-tolerated and do not relieve the whole set of symptoms. Consequently, the development of safe, efficacious and cost effective therapeutic approaches is a pressing challenge that would improve patient quality of life and alleviate the socioeconomic burden associated with the disease. The current pharmacological treatments include antibiotics, polyethylene glycol (PEG), bile salt sequestrants, antispasmodics, and antidepressants (Ford et al., 2014). However, as commented before, they often fail to optimally alleviate the symptoms. In this sense, natural products could be used to develop effective and safe therapeutic approaches (Bordbar et al., 2020).

*T. serpyllum* L. (known as wild thyme) is a perennial shrub in the Lamiaceae family native to Northern and Central Europe. Wild thyme has been broadly applied in traditional medicine for centuries and the pharmacopeias recognize its medicinal properties. *T. serpyllum* is a significant source of active ingredients with antioxidant, antimicrobial, antitumor and cytotoxic properties including essential oil, tannins, condensed tannins, basically polymers of proanthocyanidins, and gallotannins; total hydroxycinnamic acids; flavonoids; flavonones; and triterpenes (Feistel et al., 2021) which could explain its therapeutic effects. In this sense, it has been reported that *T. serpyllum* is used to treat headaches caused by colds, laryngitis, cough, upper respiratory tract infections, digestive diseases including spastic colon, meteorism, diarrhea, intestinal parasites and gastric ulcer and loss of appetite (Jarić et al., 2014). Specifically, the aerial parts of *T. serpyllum*, *Serpylli herba*, have had a great traditional use all over the world (Quave et al., 2012) as anthelmintic, antiseptic, deodorant, diaphoretic, antispasmodic, carminative, tonic, sedative and expectorant. Its beneficial health effects could be related to its content in flavonoids, phenolic acids and other active compounds, among which rosmarinic acid, a well described antioxidant, stands out (Zheng and Wang, 2001). Moreover, *Serpylli herba* extract (SHE) has recently generated a great interest for irritable bowel syndrome (IBS) management, probably due to the fact that its active compounds have also shown to preserve the intestinal barrier integrity, which is altered in patients with IBS (Barbara, 2006) and to produce intestinal anti-inflammatory effects in a variety of experimental colitis in rodents (Algieri et al., 2014).

Considering all the above, the objective of the current study was to test the beneficial effect of SHE in the experimental model of deoxycholic acid (DCA)-induced visceral pain in rats. The repetitive colorectal instillation of DCA leads to transient colonic inflammation, persistent visceral and referred mechanical hypersensitivity. That is why this model mimic the clinical manifestations human IBS (Traub et al., 2008).

## Materials and methods

### Reagents

Chemicals were obtained from Sigma Chemical (Madrid, Spain), except when mentioned specifically.

### Plant extract and drug

*Serpylli herba* aqueous extract was prepared by Finzelberg GmbH and Co. KG (Andernach, Germany) as described before (Algieri et al., 2014; Feistel et al., 2021) and supplied by PhytoLab GmbH and Co. KG (Vestenbergsgreuth, Germany). The final extract (batch TPA 47-08) consisted of 70% native extract (DERnative 4-8:1), 15% Arabic gum and 15% maltodextrin. It contained 1.8% rosmarinic acid (evaluated by HPLC), and less than 0.1% of essential oil (assessed by Clevenger apparatus), as well as less than 0.01% of thymol/carvacrol (measured by gas chromatography) (See more detailed analysis in Supplementary Material).

### Rat model of chronic post-inflammatory visceral pain induced by DCA

**FIGURE 1:**
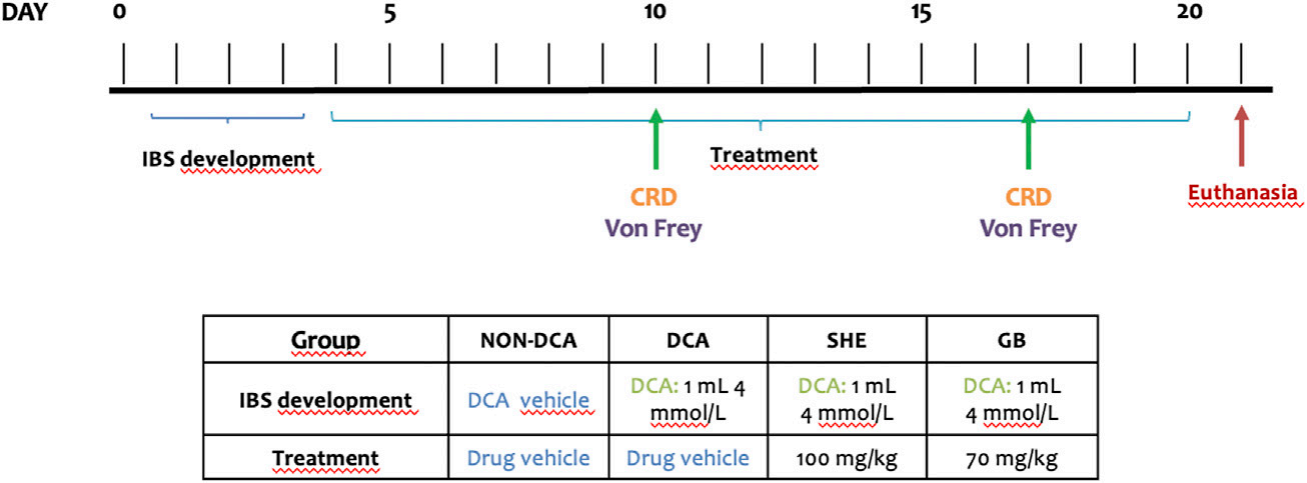
Experimental procedures. Rats were anesthetized and 1 ml of 4 mmol/L DCA was administered through the anus for three consecutive days. The drug treatments *Serpylli herba* extract (SHE) (100 mg/kg) or gabapentin (GB) (70 mg/kg) started the next day of the last DCA injection and continued for 17 days until rats were euthanized with an overdose of halothane.

Male Sprague Dawley rats (10 weeks old) with average weight of 250 ± 15 g (Janvier Labs, St Berthevin Cedex, France) were kept in a controlled environment and had *ad libitum* supply of food and water. The study was performed following the “Guide for the Care and Use of Laboratory Animals” as declared by the National Institute of Health and the protocols certified by the Ethic Committee of Laboratory Animals of the University of Granada (Granada, Spain) (17/07/2020/084). All studies concerning animals are described in agreement with the ARRIVE guidelines for reporting experiments concerning animals (Kilkenny et al., 2010; McGrath et al., 2010). Animals were in makrolon cages (5 rats/cage), maintained with a 12 h light–dark cycle and provided with *ad libitum* food and tap water in an air-conditioned atmosphere (temperature 20°C–22°C, relative humidity 45%–55%). The rats were acclimated and then randomly divided into four groups (*n* = 8). Two of them were treated with SHE (100 mg/kg) and gabapentin (70 mg/kg), respectively. Doses were chosen based on previous studies (Algieri et al., 2014). Drugs were prepared in 1 ml of carboxymethylcellulose (0.2%) and daily administered by oral gavage. An untreated DCA control group and a non-DCA control group were incorporated for reference. They were similarly administered the drug vehicle. After fasting overnight, rats were anesthetized with isoflurane and inserted a gavage needle through the anus about 6 cm to inject 1 ml of 4 mmol/L DCA in Krebs solution (in mmoles: NaCl, 122; KCl, 3.5; NaHCO3, 25; KH2PO4, 1.2; MgCl2, 1.2; pH 7.4) whilst slowly withdrawing the needle. To prevent leakage, the rats were put on a mound of bedding in a head-down position. This protocol was carried out three consecutive days. Rats in the control group underwent the same procedure but were administered 1 ml of vehicle. The drug treatments started the next day of the last DCA injection and continued for 17 days until rats were euthanized with an overdose of halothane (Figure 1). Animal body weight, water and food intake, and occurrence of diarrhea was measured every day. After euthanasia, the colon was aseptically removed, longitudinally opened and cleaned with cold saline before being minced, aliquoted and stored at −80°C till biochemical analysis.

### Gene expression analysis

Colon total RNA was isolated with NucleoZOL^®^ (Macherey-Nagel GmbH and Co., Dueren, Germany) and retrotranscribed to cDNA utilizing oligo (dT) primers (Promega, Southampton, UK). Amplification was performed using specific primers described in Table 1 mRNA relative quantitation was assessed by the 2^−ΔΔCt^ method, using glyceraldehyde-3-phosphate dehydrogenase (*Gapdh*) as a gene to normalize the data.

**TABLE 1:** qPCR primer sequences.

Gene	Sequence 5′–3′	Annealing Temperature (^°^C)	Access number
*Gapdh*	FW: CCA​TCA​CCA​TCT​TCC​AGG​AG	60	NM_017008
	RV: CCT​GCT​TCA​CCA​CCT​TCT​TG		
Ifn-γ	FW: GCC​CTC​TCT​GGC​TGT​TAC​TG	60	NM_138880.2
	RV: CCA​AGA​GGA​GGC​TCT​TTC​CT		
Il-1β	FW: GAT​CTT​TGA​AGA​AGA​GCC​CG	59	M98820
	RV: AAC​TAT​GTC​CCG​ACC​ATT​GC		
Il-6	FW: GAT​GGA​TGC​TTC​CAA​ACT​GG	60	M26744
	RV: AGG​AGA​GCA​TTG​GAA​GTT​GG		
*Muc-2*	FW: ACC​ACC​ATT​ACC​ACC​ACC​TCA​G	60	U07615
	RV: CGA​TCA​CCA​CCA​TTG​CCA​CTG		
*Muc-3*	FW: CAC​AAA​GGC​AAG​AGT​CCA​GA	60	NP_113984.1
	RV: ACT​GTC​CTT​GGT​GCT​GCT​GAA​TG		
*Cox-2*	FW: TGA​TGA​CTG​CCC​AAC​TCC​CAT​G	60	S74342
	RV: AAT​GTT​GAA​GGT​GTC​CGG​CAG​C		
*Tlr-3*	FW: GAT​TGG​CAA​GTT​ATT​CGT​C	60	XP_008769488.1
	RV: GCGGAGGCTGTTGTAGG		
*Tlr-4*	FW: AGC​TTT​GGT​CAG​TTG​GCT​CT	60	NP_062051.1
	RV: CAG​GAT​GAC​ACC​ATT​GAA​GC		
*Htr-3*	FW: CCG​GCG​GCC​CCT​CTT​CTA​T	60	NP_077370.2
	RV: GCA​AAG​TAG​CCA​GGC​GAT​TCT​CT		
*Htr-4*	FW: CAG​TTG​AAG​TTG​CCA​TCA​GC	60	NP_036985.1
	RV: CGG​CGA​ATT​GGA​GAT​GAA​CT		

### Evaluation of response to colorectal distension

The noxious visceral stimulus employed was distension with air by a flexible latex balloon inserted intra-anally (Rodríguez-Nogales et al., 2019). The response of rats to colorectal distension (CRD) was evaluated one and 2 weeks after DCA last injection. Behavioral responses to CRD were assessed in triplicate by a blinded observer after measuring the abdominal withdrawal reflex and assigning a score considering the criteria described in Table 2 (Keszthelyi et al., 2012; Zhang. et al., 2014).

**TABLE 2:** Abdominal withdrawal reflex scores.

Behavioral response	Score
Normal behavior without response	0
Brief head movement at the onset of the stimulus followed by immobility	1
Contraction of abdominal muscles	2
Lifting of the abdomen off the platform	3
Body arching and lifting of pelvic structures	4

### Determination of referred pain

Referred hyperalgesia was tested one and 2 weeks after the last injection of DCA. Von Frey filaments (Stoelting Co., Wood Dale, IL, United States) ranging from 8 g down to 1 g were applied perpendicularly to the lower abdomen. Each filament was tested by a blinded observer five times for 1-2 s and one escape response was considered as a positive reaction. The filament of the next lower force was applied till two filaments did not give a positive response.

### Quantification of *β*-Hexosaminidase release *in vitro*

The cell degranulation response was evaluated by quantifying released *β*-Hexosaminidase in culture supernatants from rat basophilic leukemia-2H3 basophils (RBL-2H3) supplied by the Cell Culture Unit of the University of Granada (Granada, Spain), which are extensively used as a mast cell model. RBL-2H3 cells (45 × 10^7^ cells/ml) were cultured for 24 h in 96-well plates at 37°C and 5% CO_2_. Next day, after washing, cells were treated with SHE at 25, 50, or 100 μg/ml or gabapentin at 5, 10, or 25 μM diluted in Tyrode’s buffer for 30 min. Then, Compound 48/80 at 50 μg/ml diluted in 100 μl of Tyrode’s buffer or 100 μl Tyrode’s buffer were added for 2 h. Tyrode’s buffer and Compound 48/80 were employed as negative and positive controls, correspondingly. Amount of *β*-Hexosaminidase in the supernatants was quantified after 90 min incubation with substrate solution (2 mM p-nitrophenyl-N-acetyl-β-D-glucosaminide in 40 mM citric acid, pH 4.5) at 37°C. The reaction was terminated by adding glycine 400 mM, pH 10.7, and optical density was determined in a microplate reader (Tecan, Männedorf, Switzerland) at 405 nm.

Data were expressed as a percentage (%) of the total *β*-Hexosaminidase, measured after lysing cells with 0.1% Triton X100 (Okochi et al., 1979).

### Statistics

In the *in vivo* and *in vitro* studies the data are expressed as the mean ± standard error of the mean (SEM) and are representative of three independent experiments. Non-parametric data were analyzed by the Kruskal–Wallis test. The von Frey data were registered as areas under the curve. For the rest of the data, multiple comparisons between groups were performed using the one-way ANOVA, followed by the Bonferroni *post hoc* test.

## Results and discussion

Nowadays the currently used drugs for IBS present variable efficacy and some of them are associated with long-term side effects (Lucak et al., 2017; Patel et al., 2021). Thus, the development of new therapeutic strategies could be of great social and clinical relevance. In this regard, alternative and complementary treatments, including herbal therapies, employed to alleviate inflammation and pain may provide some benefits (Grundmann and Yoon, 2014; Larussa et al., 2019; Kim et al., 2020). This study shows the beneficial activity of SHE in DCA-induced post-inflammatory IBS in rats.

### *Serpylli herba* extract improves visceral hypersensitivity in rats

**FIGURE 2:**
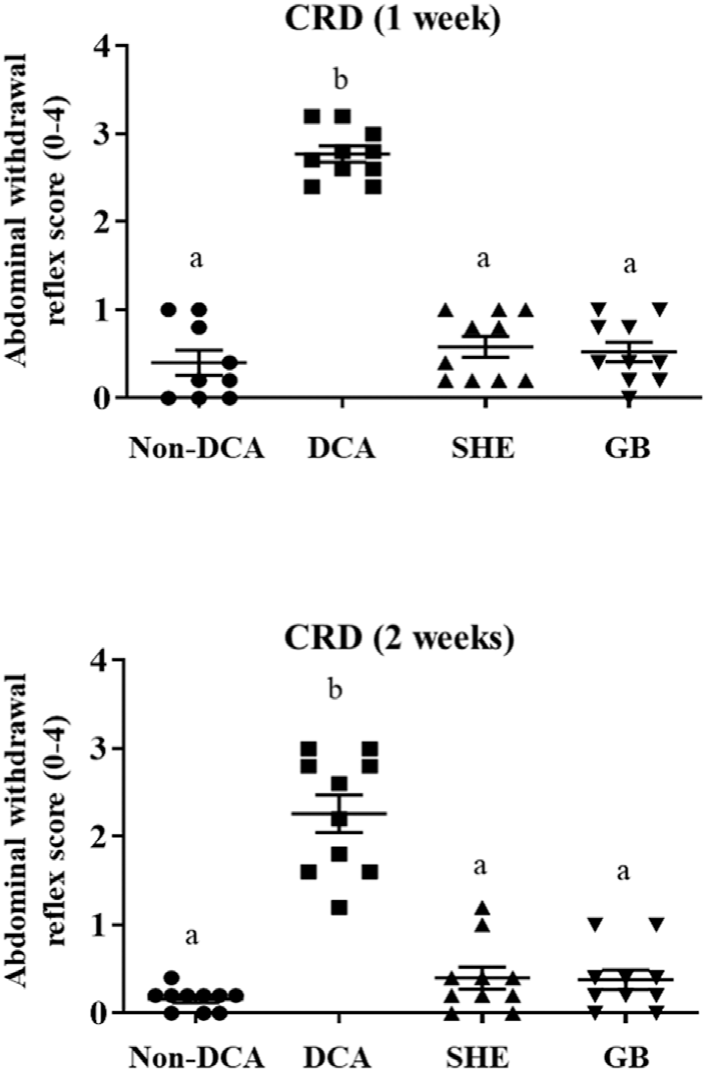
Response to colorectal distension (CRD) with a balloon catheter inflated to 60 mm Hg (20 s at 5 min intervals) was measured in triplicate one and 2 weeks after DCA intracolonic instillation in *Serpylli herba* extract (SHE) (100 mg/kg) or gabapentin (GB) (70 mg/kg) treated rats. Data are expressed as means (triplicate measurements) ± SEM (*n* = 10). Groups with different letters are statistically different (*p* < 0.05).

Visceral hypersensitivity is the most incapacitating symptom in human IBS. Different protocols have been used for decades to determine this visceral hypersensitivity. One of them is the CRD, with some resemblance to the visceral pain in the human condition (Guthrie et al., 2004). In the DCA model, this unconjugated secondary bile acid is described to increase colorectal sensitivity (Traub et al., 2008). Accordingly, our results showed higher visceral hypersensitivity to CRD (60 mm Hg), 7 and 14 days after installation of DCA, when compared to non-DCA rats (Figure 2). Interestingly, oral administration of SHE significantly lessened the CRD scores in comparison with the untreated DCA group. Of note, SHE diminished CRD scores to the same extent as gabapentin, one of the most frequently prescribed drugs against chronic neuropathic pain and for visceral hyperalgesia in human IBS (Trinkley and Nahata, 2014) (Figure 2). Indeed, other plant extracts or plant-derived natural compounds, like quercetin, have ameliorated visceral hypersensitivity in other experimental models of IBS as well as *in vitro* assays (Qin et al., 2019; Lingpeng et al., 2021). This data could support the potential future use of SHE in visceral hyperalgesia in human IBS.

### *Serpylli herba* extract supplementation ameliorates the hyperalgesia and allodynia in rats

**FIGURE 3:**
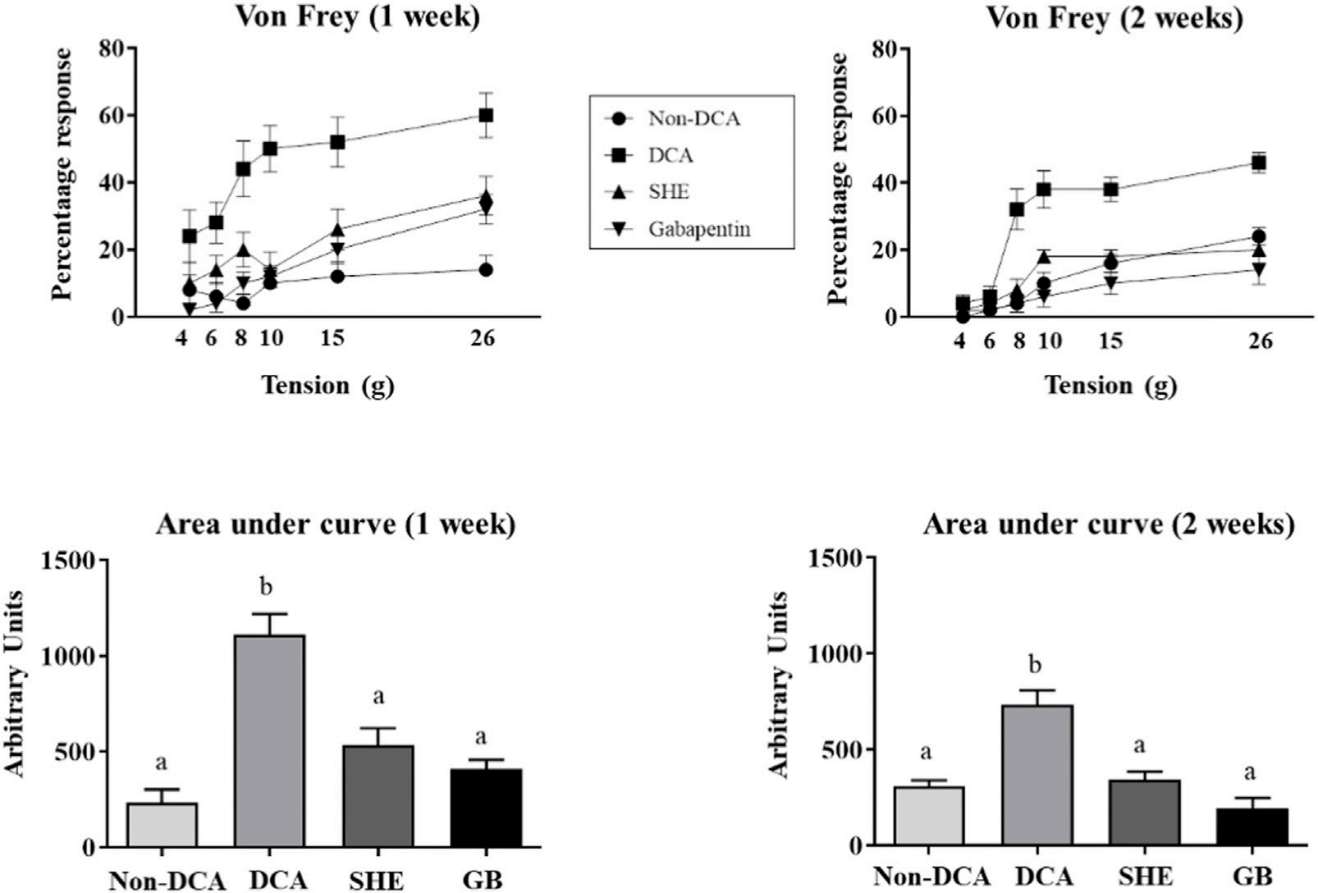
Assessment of referred hyperalgesia one and 2 weeks after DCA instillation. Von Frey filaments (1–8 g) were applied to the lower abdomen (five times for 1-2 s) of *Serpylli herba* extract (SHE) (100 mg/kg) or gabapentin (GB) (70 mg/kg) treated rats. Referred hyperalgesia was estimated considering percentage of response to von Frey filaments and area under the curve (AUC) was calculated. Data are expressed as means ± SEM (*n* = 10). Groups with different letters are statistically different (*p* < 0.05).

It has been previously reported that intracolonic injection of DCA (4 mM) causes mild inflammation in rats, typified by visceral pain for at least 28 days (McGrath et al., 2010). Accordingly, evaluation of the referred pain generated in the lower abdomen with von Frey filaments one and 2 weeks after DCA instillation showed a lower threshold for the response in the untreated DCA control group than the non-DCA control group, indicating an elevated sensitivity (Figure 3). Moreover, the percentages of responses were significantly increased at all the pressures tested, being more intense at week one than at week two (Figure 3). The administration of SHE or gabapentin significantly reduced the nociceptive score after the mechanical stimulation with von Frey filaments (from 6 to 26 g) (Figure 3). It is important to highlight that, although other plant extracts have been described as potential adjuvant therapies for allodynia and hyperalgesia in experimentally-induced neuropathic pain (Amoateng et al., 2015; Hosea Azi Iliya et al., 2016; Shahid et al., 2017; Xie et al., 2018), this is the first study that shows the ability of SHE to improve mechanical somatic hyperalgesia and allodynia caused by DCA in rats, at the same extent as gabapentin, a widely used drug for visceral pain in human IBS (Trinkley and Nahata, 2014) that has also supported by clinical studies, in which symptoms of abdominal pain, hyperalgesis, urgency, and bloating were significantly improved (Lee et al., 2005; Houghton et al., 2007). In this sense, gabapentin treatment has shown ability to reduce rectal sensory thresholds through attenuating rectal sensitivity to distension and enhancing rectal compliance in diarrhoea-predominant IBS patients (Lee et al., 2005; Zhang, et al., 2014; Moloney et al., 2015). Studies have indicated that the pain-relieving effect of gabapentin is through binding with α2δ-1, an auxiliary subunit of voltage gated calcium channels (Houghton et al., 2007).

### *Serpylli herba* extract diminishes the IBS-associated gut inflammatory process

**FIGURE 4:**
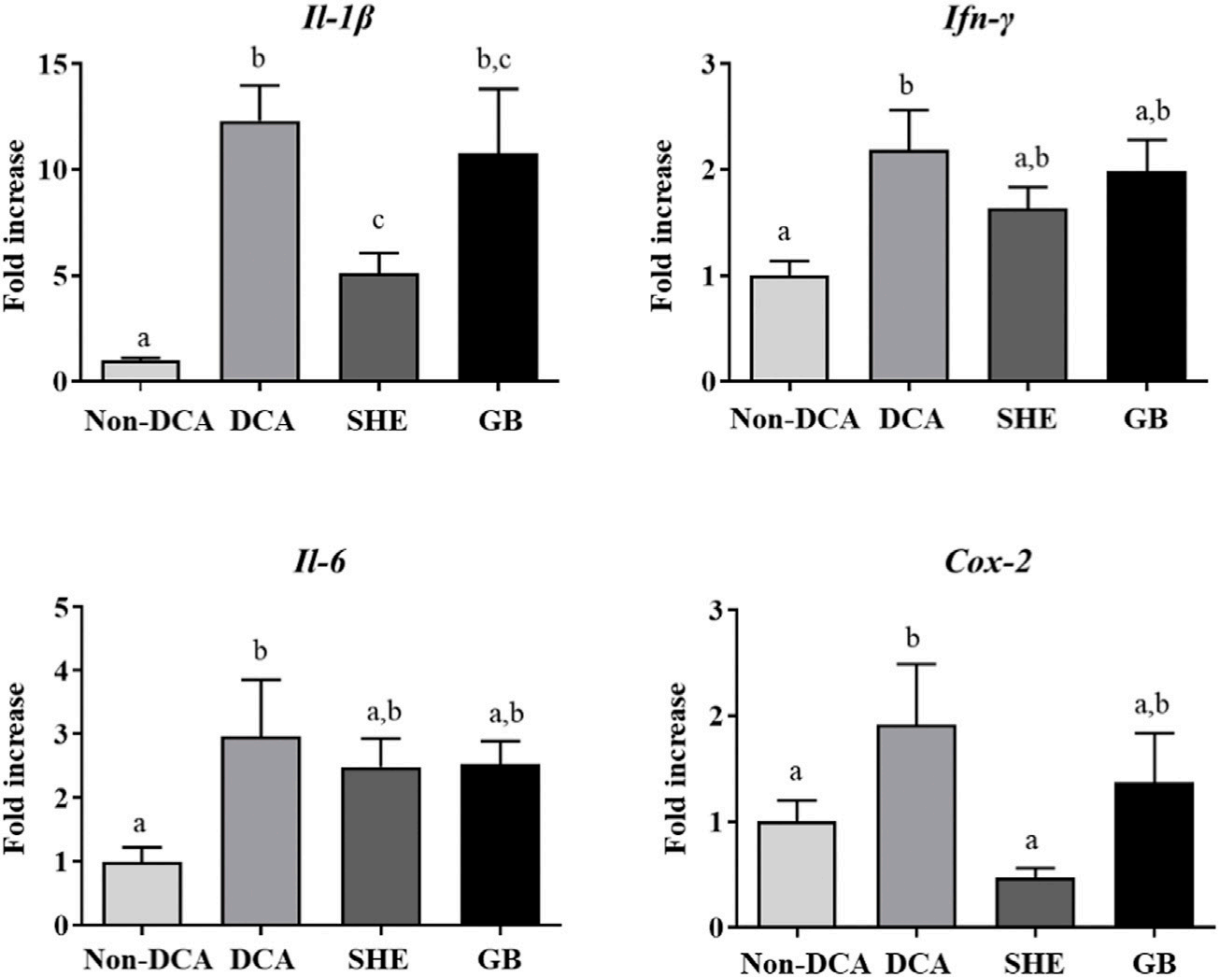
Impact of *Serpylli herba* extract (SHE) (100 mg/kg) or gabapentin (GB) (70 mg/kg) treatment on inflammatory status: gene expression of Il-1β, Ifn-γ, Il-6, and *Cox-2*. Data are expressed as means ± SEM (*n* = 10). Groups with different letters are statistically different (*p* < 0.05).

The colonic installation of DCA to rats also induced a short-lasting low-grade inflammatory response in the large intestine (Traub et al., 2008). However, an alteration of the immune response was still obvious at the end of the experiment, 3 weeks after the first administration of DCA. The expression of the different mediators, including Il-1β, Il-6, Ifn-γ and *Cox-2*, was higher in the IBS control group without treatment compared to non-DCA control (Figure 4), the same as it has been previously reported, both in experimental models and in humans (Algieri et al., 2014). The expression of Il-1β and *Cox-2* was significantly reduced after the administration of SHE, when compared to the IBS control rats (Figure 4), similarly to effect obtained with gabapentin (Figure 4). The inflammatory implications in IBS are controversial (Martin-Viñas and Quigley, 2016) but it has been reported that the activation of the immune cells is involved in the pathology of IBS (Barbara et al., 2011). Stimulated mast cells appear to be key for the release of various biologically active compounds, such as those involved in the degranulation pathway, cytokines (IL-1β, IL-6, and IFN-γ), and metabolites derived from membrane arachidonic acid, including prostaglandins derived from COX-2 induction (Gwee et al., 2003). The ability of SHE treatment to reduce the colonic expression of *Cox-2* might downregulate prostaglandin production and secretion, typically associated with these eicosanoids. This could ameliorate the hyperalgesia, given the ability of these mediators to lower the activation level of sensory afferents *via* activation of EP1 receptors (Sarkar et al., 2003). As commented above, it is still unknown why the expression of these cytokines is upregulated, but it could be associated with a disproportionate immune activation by the gut microorganisms, involving a mucosal barrier dysfunction, and/or stress-induced stimulation of the immune response. Moreover, SHE administration also reduced the colonic expression of Il-1β, Il-6, and Ifn-γ, effects previously seen in experimental colitis in rodents (Algieri et al., 2014). Therefore, our findings support that the immunomodulatory abilities of SHE participate in its beneficial effect.

### *Serpylli herba* extract treatment restores the altered gut epithelial barrier induced by DCA in rats

**FIGURE 5:**
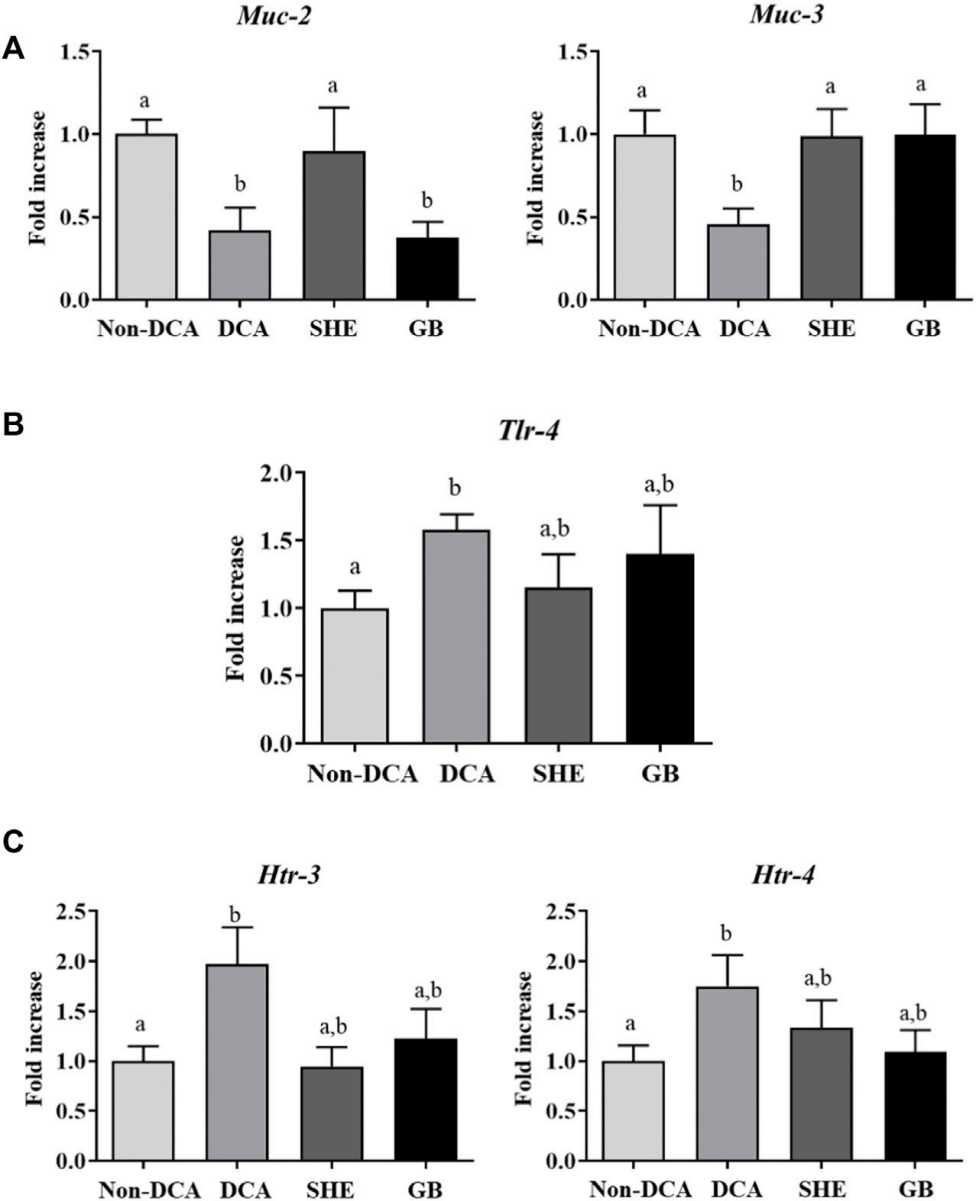
Effects of *Serpylli herba* extract (SHE) (100 mg/kg) or gabapentin (GB) (70 mg/kg) administration on **(A)** colonic gene expression of *Muc-2* and *Muc-3*; **(B)** colonic gene expression of *Tlr-4* and **(C)** *Htr-3* and *Htr-4* expression. Data are expressed as means ± SEM (*n* = 10). Groups with different letters are statistically different (*p* < 0.05).

Many studies have reported the correlation between compromised epithelial barrier function and impaired immune stimulation and gut malfunction in human IBS (Piche, 2014; Shukla et al., 2018). Our study coincides with these observations since IBS rats showed a decreased expression of the mucins *Muc-2* and *Muc-3,* the major glycoproteins in the mucus protective barrier of the gastrointestinal surface, compared to the saline group (Figure 5A). This modified expression of mucins has also been shown other experimental models of IBS in rats, including the water avoidance-stress and the neonatal maternal separation (Da Silva et al., 2014; Barouei et al., 2015). Remarkably, SHE was able to normalize the expression of both mucins, thus promoting epithelial barrier restoration. Similarly, numerous researchers have evidenced the key role of several compounds (i.e., phenolic compounds) in restoring the damaged gut barrier *in vivo* animal models of intestinal inflammation, however, their mechanism of action is still unknown (Bernardi et al., 2020; Sandoval-Ramírez et al., 2021). Nevertheless, different mechanisms may be involved, including: 1) inhibition of pro-inflammatory molecules; 2) increased expression of tight-junction proteins, and 3) enhancement of intracellular antioxidant activity. Consequently, summing up the current results and others obtained in various experimental models of intestinal inflammation, SHE could be considered to improve the intestinal mucosal barrier integrity through the mechanisms described above. On the contrary, gabapentin only restored *Muc-3* expression which may imply that this drug might act through other mechanisms (Figure 5A).

The imbalance in the mucosal barrier and the immune response previously mentioned may arise from a dysregulation of the intestinal microbiota, which could favor aberrant immune responses, gut inflammation (Shulman et al., 2014) and expression of toll-like receptors (TLRs). These are Pattern-recognition receptors, key for the mucosal immune response in human IBS (Belmonte et al., 2012), trigger the innate immune response through the production of cytokines and chemokines (Brint et al., 2011). TLR-4 recognizes lipopolysaccharides (LPS) of Gram-negative bacteria, and it is widely known that the administration of Gram-negative bacterial LPS to human volunteers induces the secretion of pro-inflammatory mediators, including TNF and IL-6, in addition to the fact that patients with diarrhea-predominant IBS display increased LPS serum levels (Dlugosz et al., 2015). Indeed, in the present study, the DCA group manifested an increased expression of colonic *Tlr-4* mRNA compared with the untreated control group, while lower expression rates were observed in the SHE-treated group (Figure 5B), which may be explained considering the effect shown in the mucosal barrier and the inflammatory status. This result reveals that the enhancement in the barrier function may be linked to a modulation of the response in the mucosa to specific microbial components of the gut lumen, preventing diverse stimuli that may be related to the visceral hyperalgesia that takes place in experimentally induced IBS.

Similarly, alterations in 5-HT homeostasis are pivotal in IBS. In fact, different 5-HT agents have been utilized therapeutically in IBS patients in whom 5-HT_3_ receptor antagonists have shown an improvement of the gastrointestinal symptoms, lowering stool frequency, urgency, and abdominal discomfort while augmenting stool consistency (Lacy, 2016). Thus, 5-HT receptor 4 (*Htr-4*) and 3 (*Htr-3*) expressions have been described to be determining in the pathology of visceral hypersensitivity through the release of several neurotransmitters and the progress of IBS (Yan et al., 2012; Fu et al., 2019). Consequently, when the expression of these receptors was assessed in the present study, the IBS group displayed a double-fold increase in comparison with the control group (Figure 5C), which corroborates previous mentioned observations. Notably, both SHE and gabapentin treatment managed to reestablish the expression of both 5-HT receptors (Figure 5C), which suggests that this mechanism of action could be involved in their beneficial effect in the DCA experimental model.

### *Serpylli herba* supplementation weakens the mast cell degranulation *in vitro*

**FIGURE 6:**
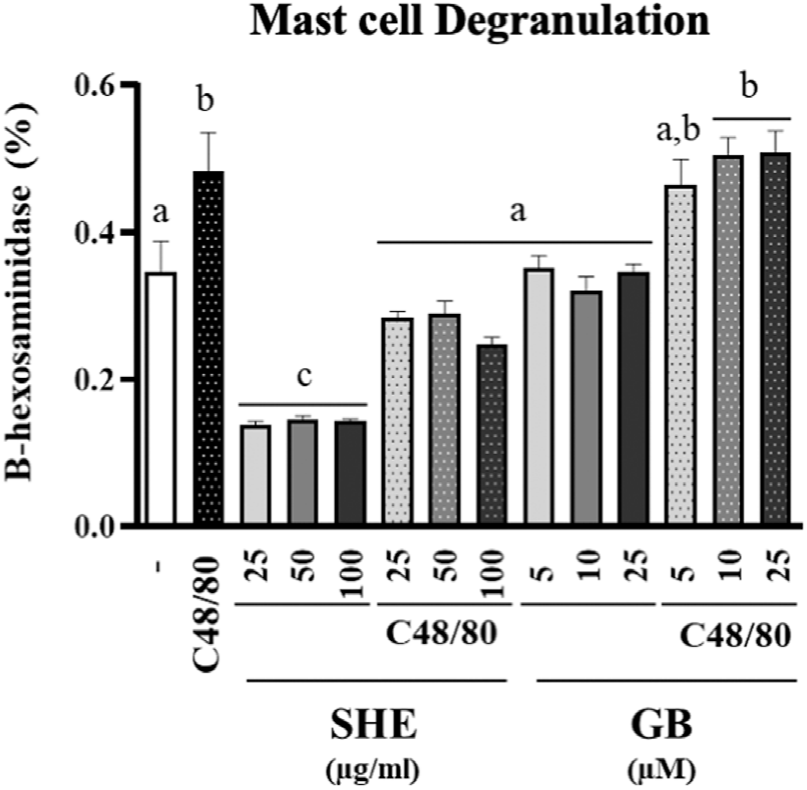
Determination of β-Hexosaminidase production in RBL-2H3 cells. Cells were incubated with *Serpylli herba* extract (SHE) (25, 50, and 100 μg/ml) or gabapentin (GB) (5, 10, and 25 μM) for 30 min and then stimulated with compound C48/80 or vehicle. Experiment was performed in triplicate. Data are expressed as means ± SEM. Groups with different letters are statistically different (*p* < 0.05).

To better understand the effect of SHE in the IBS experimental model induced by DCA, we assessed the activity of SHE in a stimulated RBL-2H3 cell line. RBL-2H3 cell line shows similar features with basophils and mast cells (Passante and Frankish, 2009). Mast cells are crucial in the pathophysiology of functional gut conditions, mainly in IBS, being considered as a pharmacological target for its management. Moreover, mast cells show other functions, including modulation of permeability, secretion, peristalsis, nociception, immunity, and angiogenesis (Krystel-Whittemore et al., 2015). Recently, these cells have being paid much attention in IBS because of their sensor and effector function (Lee and Lee, 2016). In fact, a significant greater infiltration of these cells has been observed in the intestinal mucosa in IBS in comparison with non-IBS controls (Park et al., 2006; Guilarte et al., 2007; Sohn et al., 2014). Moreover, an increment in their activity has been described (Cremon et al., 2009). Correspondingly, the data obtained with C48/80-stimulated RBL-2H3 cells indicated that only the SHE supplementation (25, 50, and 100 μg/ml) significantly inhibited β-Hexosaminidase production induced by C48/80 (Figure 6) in comparison to non-stimulated cells, without affecting cell viability (data not shown). Other plant extracts containing flavonoids (such as luteolin and rutin) in their composition have been described to reduce the expression of β-Hexosaminidase *in vitro*, both in human and rodent mast cells (Park et al., 2008; Seelinger et al., 2008; Hagenlocher et al., 2017). These results reveal the highly relevant and novel mechanism of action of the SHE administration; its beneficial effect can be ascribed to its capacity to suppress the release of β-Hexosaminidase and consequently to modulate the mast cell degranulation.

## Conclusion

This study showed the beneficial effects of SHE on an experimental IBS model. SHE lessened DCA-induced visceral hyperalgia and referred pain. Additionally, SHE improved the immune status of the rats, lowering the expression of pro-inflammatory mediators that could play a part in the visceral analgesia achieved, and repairing the gut epithelial barrier integrity. One of the mechanisms of action implicated in the beneficial effect of SHE in the DCA experimental model seems to be mediated by the serotonin pathway. Furthermore, this study suggests, for the first time, novel mechanisms of action that might be involved, including inhibition of mast cell degranulation. Consequently, it would be worthy to deeper explore the administration of SHE in the prevention and/or treatment of IBS.

## Data Availability

The original contributions presented in the study are included in the article/Supplementary Material, further inquiries can be directed to the corresponding authors.

## Abbreviations

CR, colorectal distension; DCA, deoxycholic acid; *Gapdh*, glyceraldehyde-3-phosphate dehydrogenase; IBS, irritable bowel syndrome; LPS, lipopolysaccharide; PEG, polyethylene glycol; RBL-2H3, rat basophilic leukemia-2H3 basophils; SHE, *Serpylli herba* extract; Ta, annealing temperature; TLRs, toll-like receptors.
