# Supplementary material for: Intestinal anti-inflammatory and visceral analgesic effects of a Serpylli herba extract in an experimental model of irritable bowel syndrome in rats
Source: Front Pharmacol. 2022 Sep 2;13:967644. doi: 10.3389/fphar.2022.967644 (PMC9479127; doi:10.3389/fphar.2022.967644)
Supplement: Supplementary file 1 [file DataSheet1.docx]

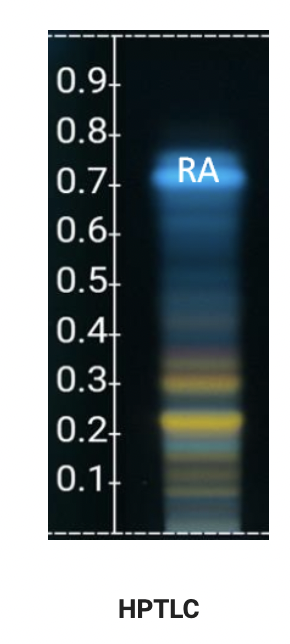


Figure S1. High-Performance Thin Layer Chromatography (HPTLC). HPTLC acc. to Ph.Eur. 9.0: TLC silica gel plate with formic acid / water / ethyl acetate (1:1:15 V/V/V) as mobile phase, Naturstoff reagent (DPBA), UV 365 nm; RA, rosmarinic acid). The main phenolic acid in the extracts was rosmarinic acid, followed by salvianolic acid I, salvianolic acid K isomer and caffeic acid, whereas chlorogenic acid was detected only in traces; the dominant flavonoids were luteolin 7-O-glucuronide and 6-hydroxyluteolin 7-Oglucoside, while the contents of apigenin glucuronide and 6,8-di-C-glucosylapigenin were significantly lower.


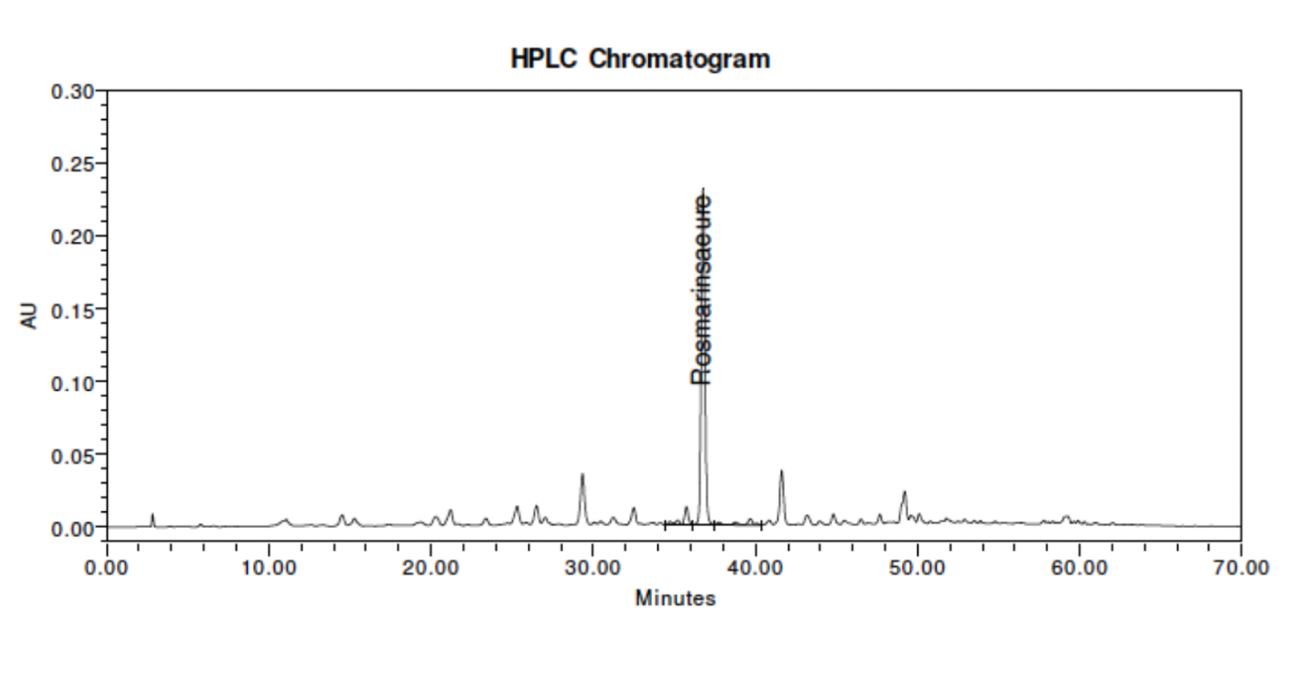
Figure S2. Determination of *Serpilly Herba* aqueous extract by High-performance liquid chromatography (HPLC).
